# Gas-Plasma-Activated Water Impact on Photo-Dependent Dormancy Mechanisms in *Nicotiana tabacum* Seeds

**DOI:** 10.3390/ijms23126709

**Published:** 2022-06-16

**Authors:** Giles Grainge, Kazumi Nakabayashi, Felipe Iza, Gerhard Leubner-Metzger, Tina Steinbrecher

**Affiliations:** 1 Department of Biological Sciences, Royal Holloway University of London, Egham TW20 0EX, UK; giles.grainge.2010@live.rhul.ac.uk (G.G.); kazumi.nakabayashi@rhul.ac.uk (K.N.); gerhard.leubner@rhul.ac.uk (G.L.-M.); 2 Wolfson School of Mechanical, Electrical and Manufacturing Engineering, Loughborough University, Leicestershire LE11 3TU, UK; f.iza@lboro.ac.uk; 3 Division of Advanced Nuclear Engineering, Pohang University of Science and Technology (POSTECH), Pohang 790-784, Korea; 4 Laboratory of Growth Regulators, Institute of Experimental Botany, Czech Academy of Sciences and Faculty of Science, Palacký University, CZ-78371 Olomouc, Czech Republic

**Keywords:** seed dormancy, gas-plasma-activated water, tobacco, germination, photo-dependent dormancy, gibberellin oxidase

## Abstract

Seeds sense temperature, nutrient levels and light conditions to inform decision making on the timing of germination. Limited light availability for photoblastic species results in irregular germination timing and losses of population germination percentage. Seed industries are therefore looking for interventions to mitigate this risk. A growing area of research is water treated with gas plasma (GPAW), in which the formed solution is a complex consisting of reactive oxygen and nitrogen species. Gas plasma technology is widely used for sterilisation and is an emerging technology in the food processing industry. The use of the GPAW on seeds has previously led to an increase in germination performance, often attributed to bolstered antioxidant defence mechanisms. However, there is a limited understanding of how the solution may influence the mechanisms that govern seed dormancy and whether photoreceptor-driven germination mechanisms are affected. In our work, we studied how GPAW can influence the mechanisms that govern photo-dependent dormancy, isolating the effects at low fluence response (LFR) and very low fluence response (VLFR). The two defined light intensity thresholds affect germination through different phytochrome photoreceptors, PHYB and PHYA, respectively; we found that GPAW showed a significant increase in population germination percentage under VLFR and further described how each treatment affects key physiological regulators.

## 1. Introduction

The seed’s role is to disperse and protect the embryo before determining an appropriate time to germinate in favourable conditions [1]. Seeds achieve this by sensing abiotic information, such as nutrients, temperature and light, to control the seasonal timing of germination in the natural environment. Dormancy is an intrinsic seed characteristic that defines the environmental conditions required for germination, with light conditions providing both temporal (e.g., seasons) and spatial (e.g., soil depth) information [2,3]. Seeds that require either light or darkness to germinate are considered to have photo-dependent dormancy blocks. When deeply buried seeds attempt germination, seedlings exhaust their energetic compounds before the embryonic leaves can reach the surface to initiate photosynthesis [4]. Therefore, photo-dependent dormancy is an evolutionary trait that is essential for ensuring plant survival and the ability to adapt to new environments. It is also a critical and often limiting factor for seedling establishment and yield in agricultural production. Light is a crucial environmental cue that plays a fundamental role in plant development. Plants perceive light through photoreceptors, of which there are five groups: phytochromes, cryptochromes, phototropins, UV light receptors (such as UVR8) and zeitlupes [5,6,7]. The primary receptors that regulate seed germination are the phytochromes mediating red (R) and far-red (FR) responses [8]. These proteins can change structure and function in a photo-reversible manner in response to the light’s wavelength and intensity. Arabidopsis has five phytochromes representing two physiologically defined groups, of which two—PHYA and PHYB—are most important to regulate seed germination. These phytochromes are also found in other species that display photo-dependent dormancy, including *Nicotiana tabacum.* PHYA is the sole member of the ‘light-labile’ phytochrome group known to mediate light effects at extremely low light intensities (photon fluences). These are described as a very low fluence response (VLFR), which is irreversibly activated through exposure to both red and far-red light [9,10]. PHYB is the member of the ‘light-stable’ phytochrome group representing a different mode of action. It requires higher light intensities (LFR, low fluence response) to reversibly switch between a bioactive and non-active form across R and FR light shifts [11,12]. Therefore, the two phytochromes regulate seed germination through distinct mechanisms: in the presence of far-red light, PHYB is inactivated whilst a sufficiently high proportion of PHYA remains active to trigger VLFR [13]. 

These photoreceptors are integrated within the seed’s molecular network, which regulates the phytohormone ABA’s and GA’s concentration balance [14]. Photo-dependent dormancy alleviation is associated with a decrease in ABA content and sensitivity, as well as with an increase in GA sensitivity and concentration [2]. There are a number of key regulatory genes that drive this shift in phytohormone balance. The phytochrome-interacting factor PIF1 is negatively regulated by bio-active PHYB and is known to maintain seed dormancy by influencing the mechanisms that dictate ABA and GA balance [15]. GA 20-oxidases (GA20ox) and GA 3-oxidases (GA3ox) are vital regulatory catalysts in the pathway that accumulate the bio-active form of GA that can be further catabolised through oxidation by GA 2-oxidase (GA2ox), an enzyme understood to be a critical negative regulator of bio-active GA accumulation [16].

It is known that the PHYA-dependent control of germination and the PHYB-dependent control of germination are spatially and temporally separated in the endosperm and embryo, respectively. It is also known that they occur at different stages of germination, and that PHYB predominantly triggers red/far-red light-reversible seed germination and PHYA mediates distinct VLFR in red and far-red light [9,10,13,17]. However, very little is known of the effect of VLFR in germination in contrast to LFR. In *Datura ferox* seeds, VLFR triggers the activation of endosperm-weakening-related enzymes, endo-beta-mannanase and beta-mannosidase [18]. It also up-regulates the *GA3ox* gene, suggesting that the promotion of germination by VLFR is associated with an increase in the synthesis of active gibberellins. Barros-Galvão et al. [17] recently described a unique mechanism in which PHYA activation promoted *ABI4*, which in turn repressed *NCED6* and *NCED9* in *Arabidopsis thaliana*, thus stunting ABA accumulation. The downstream genes of the phytohormone pathways, which alleviate the mechanical resistance imposed on the micropylar endosperm that dictates germination timing, are strong indicators of dormancy release [19]. Genes for expansins (*EXPA*) and xyloglucanendo-transglycosylase/hydrolases (*XTH*) are up-regulated within the micropylar endosperm of non-dormant seeds through a GA-induced mechanism [20]. PIF1, which is suppressed by bio-active PHYB, represses *EXPA* and *XTH* expression, thus preventing germination.

Gas plasma technology is a growing sector that is receiving increasing attention for its applications in sterilisation and agriculture, where it has been shown to enhance seed germination and plant development [21,22]. Among other physical (e.g., radiation and electric water activation) and chemical (nutrients, fungicide, and insecticide) seed treatment techniques [23,24,25], gas-plasma-activated water (GPAW) is a sustainable emerging seed treatment technology. GPAW solutions are produced by exposing water to a gas plasma that is sustained by applying high voltage electrical energy to a background gas. This results in the ionisation of the gas and the synthesis of a myriad of charged ions, reactive species, and free electrons. Plasma produces species diffuse and further react in H_2_O, creating GPAW with a chemistry that depends on the original gas mixture used in the plasma and the preparation conditions [26,27]. Using oxygen and nitrogen as part of the carrier gas for the plasma results in the formation of signalling reactive oxygen and nitrogen species that include transient species such as hydroxyl radicals (OH·), nitrogen dioxide (·NO_2_), and nitric oxide (·NO), as well as longer-life chemical products such as hydrogen peroxide (H_2_O_2_), nitrate (NO_3_^−^), and nitrite (NO_2_^−^). Furthermore, removing nitrogen from the gas mixture and replacing it with an inert carrier gas, such as helium or argon, suppresses the synthesis of nitrogen-based species and enhances the production of reactive oxygen species [28].

To date, the majority of research on how non-thermal atmospheric gas plasma improves seeds germination performance has been focused on direct plasma treatment [21]. More limited are publications that have focused on the application of GPAW to treat commercial crop and vegetable seed species, though a number of effects have already been noted. The use of GPAW has been shown to increase germination speed in several species; influence antioxidant machinery, resulting in higher levels of stress tolerance; and effectively disinfect the surface of seeds; which is a previously established application of the technology in other industries such as food packaging preparation, medicine, and water treatment [29,30]. Little work has been conducted to fully understand how GPAW influences germination, and only few researchers have looked into the underlying mechanisms [31]. Researchers have presented evidence on dormancy alleviation in *A. thaliana* [31], as well as some evidence indicating the influence on the antioxidant machinery in rice [32]. However, there is still a knowledge gap on how GPAW can influence dormancy regulation, and it is unknown how these effects take place under different light conditions.

In this study, we exploited a proven model system of photo-dependent dormancy, *Nicotiana tabacum* [33], to investigate the effects of GPAW on the germination of dormant seeds across a range of light conditions. GPAW samples created with an air plasma and a helium–oxygen mixture were considered in the study, and the underlying molecular mechanisms triggered by GPAW that affect the photo-dependent dormancy molecular networks were revealed.

## 2. Results

### 2.1. Impact of Gas-Plasma-Activated Water (GPAW) on Photo-Dependent Dormancy

**Figure 1:**
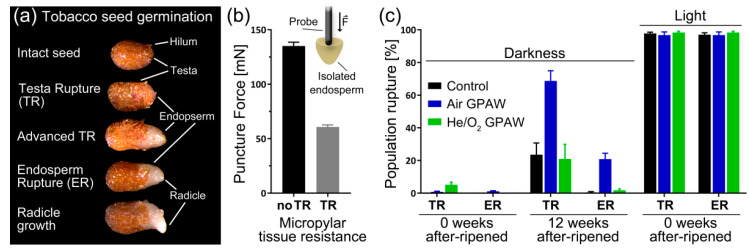
*Nicotiana tabacum* seed germination. (**a**) Different stages of the germination process showing testa rupture (TR), endosperm rupture (ER) and radicle elongation. (**b**) Force needed to puncture the micropylar part of the endosperm before and after testa rupture. (**c**) Comparison of 0 and 12 week after-ripened (33% RH and 20 °C) seeds’ testa rupture (TR) and endosperm rupture (ER) (7 days after imbibition in both dark and light germinated in water or gas-plasma-activated water (GPAW)).

*Nicotiana tabacum* has a two-step germination process, in which the ruptures of the testa (seed coat) and the endosperm are separate events (Figure 1a). During the germination process, the micropylar endosperm weakens after visible testa rupture occurs. The force needed to puncture the endosperm drops from 135 to 60.8 mN (Figure 1b). The freshly harvested dormant *N. tabacum* cv. Havana 425 seeds used in this experiment were positively photo-dependent in germination in full light, as near 100% population germination was recorded (Figure 1c); however, without any after-ripening storage (0 weeks of after-ripening), no germination was evident in complete darkness. Interestingly, neither treatment, with Air GPAW of He/O_2_ GPAW, released primary dormancy in complete darkness without after-ripening storage (Figure 1c). However, the after-ripening of *N. tabacum* seeds for 12 weeks lead to a significant response in darkness to Air GPAW, as testa rupture (TR) increased by 45% (*p* < 0.0001) and endosperm rupture (ER) increased by 20% (*p* = 0.003) compared to the after-ripened control seeds and He/O_2_ GPAW-treated seeds (Figure 1c).

**Figure 2:**
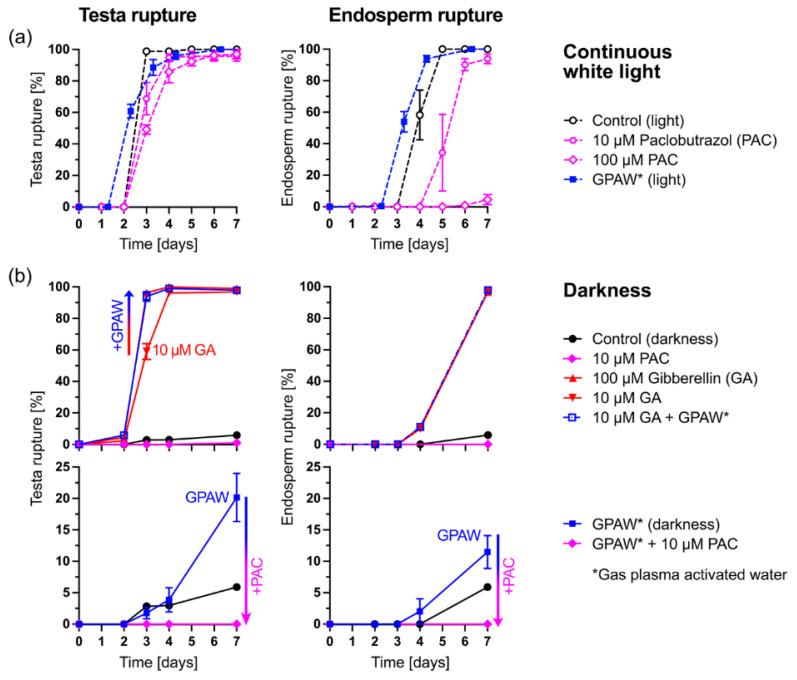
The effects of gas-plasma-activated water (Air GPAW) and GA biosynthesis inhibition on the time courses of *Nicotiana tabacum* testa rupture (TR) and endosperm rupture (ER). (**a**) Effect of the GA biosynthesis inhibitor paclobutrazol (PAC) and GPAW on TR and ER in continuous white light (ca. 111 µmol m^−2^ s^−1^). (**b**) The effects of GPAW, GA and PAC treatment on the TR and ER of tobacco seeds imbibed in darkness. Results are shown as mean ± SEM of three petri dishes each with 40 seeds imbibed at 20 °C.

Seed dormancy release, endosperm weakening and the germination of positively photoblastic seeds including *N. tabacum* and *A. thaliana* are promoted by light-induced GA biosynthesis and signalling [2,6,7,8,9,10,11,12,13,14,15,16,17,18,19,20]. Scoring tobacco TR and ER (Figure 1a) over time is therefore an excellent system to study the possible roles of GPAW in the interaction between GA and light in these processes. Figure 2 shows the results for a partially after-ripened tobacco seed batch that was characterised by full germination in continuous light (Figure 2a) but no TR and ER upon imbibition in darkness (Figure 2b). The treatment of light-imbibed tobacco seeds with the classical GA biosynthesis inhibitor paclobutrazol (PAC) slightly delayed TR but strongly inhibited GA-dependent ER in a dose-dependent manner (Figure 2a). In agreement with a role of GPAW in affecting GA biosynthesis and/or signalling, treatment with Air GPAW slightly promoted the TR and ER of light-imbibed tobacco seeds (Figure 2a). 

The treatment of dark-imbibed tobacco seeds with GA released the block to TR in a dose-dependent manner (Figure 2b). The combined treatment of 10 µM GA plus GPAW enhanced TR compared to 10 µM GA alone and delivered a temporal pattern similar to 100 µM GA. Furthermore, the treatment of the dark-imbibed tobacco seeds with GPAW resulted in a TR of ca. 20% of the population (Figure 2b). However, this effect of the GPAW was completely abolished by the combined treatment of GPAW with PAC, which provides evidence that GPAW acts, at least in part, by replacing the light requirement for the GA biosynthesis induction of this seed population. This finding extends earlier work in *A. thaliana* that demonstrated that GPAW targets GA metabolism and downstream genes to release seed dormancy and promote germination [31].

#### 2.1.1. Air GPAW Impact on *N. tabacum* Photo-Dependent Dormancy Alleviation

**Figure 3:**
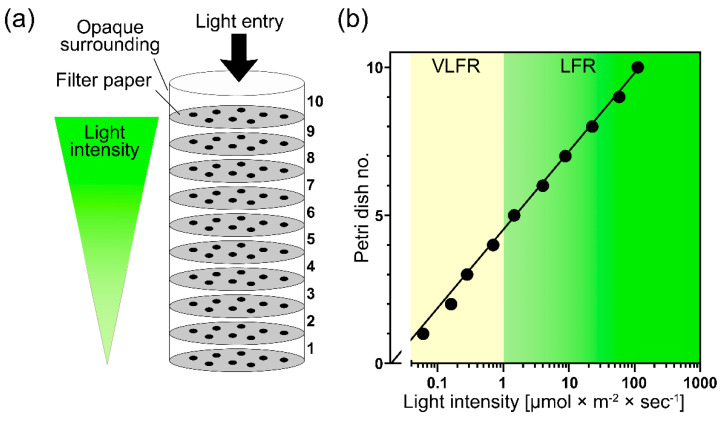
Experimental design and continuous white-light intensity values of the (**a**) ‘light filter tube’ consisting of 10 stacked petri dishes used to assess changes in light dependency for germination of dormant *Nicotiana tabacum* seeds. The dishes were wrapped in opaque material, limiting light access to the top of the petri dishes and generating an exponential decreasing light intensity through each layer. (**b**) White-light intensities in the petri dish stack are expressed as photon fluence rate (µmol m^−2^ s^−1^). The very low fluence response range (VLFR) is shaded yellow (<1 µmol m^−2^ s^−1^), and the low fluence response (LFR) range is on the right segment of the graph (>1 µmol m^−2^ s^−1^).

To further investigate the light–darkness effects on seed dormancy combined with GPAW treatments, we compared the germination of freshly harvested dormant *N. tabacum* seeds in complete darkness with an exponential increase in white light (continuous WL) intensity using the system described in Figure 3. To score germination within a light gradient, a ‘light filter tube’ was constructed, as shown in Figure 3a. The construction consisted of ten 6 cm diameter petri dishes placed on top of each other containing two filter papers, 1.5 mL of treatment solution and *N. tabacum* seeds intended for germination testing. The stack of petri dishes was wrapped in opaque black card to limit light access to the top petri dish. Each petri dish filtered out 55.8% ± 2.4 of photosynthetic active radiation, creating an exponential decay of light availability through each petri dish layer (Figure 3b).

**Figure 4:**
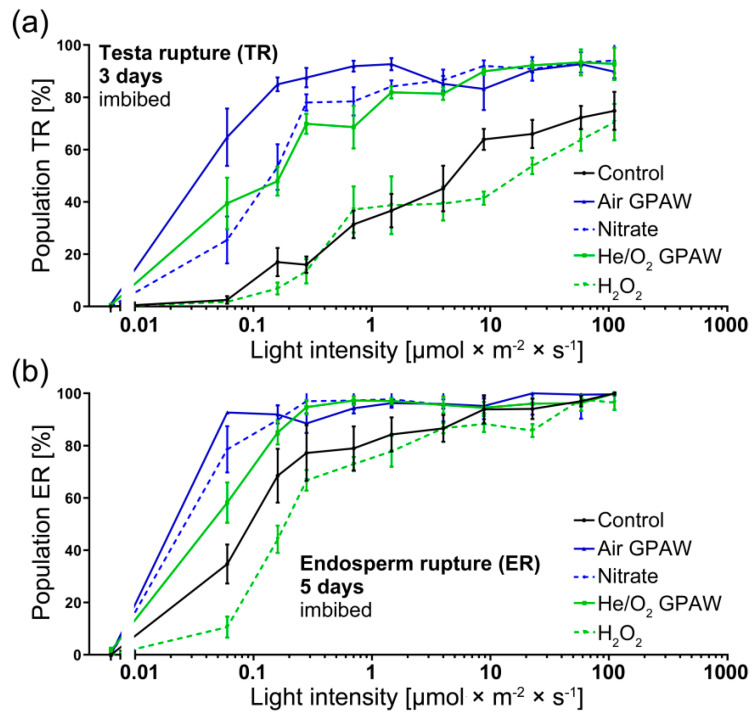
*Nicotiana tabacum* seed germination under various continuous white-light regimes with and without treatments. (**a**) TR (3 days imbibition) and (**b**) ER (5 days imbibition) percentage of dormant *N. tabacum* in relationship to light availability plotted on a logarithmic x axis. There were 4 treatment groups; Air GPAW, He/O_2_ GPAW, Nitrate (5 mM KNO_3_^−^) and H_2_O_2_ (300 µM); control seeds were imbibed in water.

Increasing light intensity, corresponding to values typical for very low fluence response (VLFR) and low fluence response (LFR), caused significant differences in TR and ER percentages for both GPAW treatments when compared to the control (Figure 4). Both GPAW treatments lead to increased TR (3 days post imbibition) in contrast to the control (*p* < 0.05) (Figure 2a). For ER (5 days post imbibition), all treatments in the VLFR range, except the direct H_2_O_2_ treatment, resulted in an increased ER compared to the control (Figure 4b).

Looking at Air GPAW in detail, TR was significantly increased 3 days post imbibition for all light conditions considered in the study (Figure 4a). Under VLFR conditions (<1 µmol m^−2^ s^−1^), Air GPAW treatment resulted in a mean percentage differences with respect to the control of 62.2%, 68.0%, 71.6% and 60.5% at 0.06, 0.16, 0.28, and 0.7 µmol m^−2^ s^−1^, respectively. Across the LFR light intensity range, Air GPAW also showed significant improvements at 1.5, 4, 8.8, and 58.1 µmol m^−2^ s^−1^ that, respectively, led to significant 56.0%, 40.01%, 19.3%, and 20.4% mean differences with respect to the control. Air GPAW contains a significant concentration of nitrates, which could contribute to the observed response. However, chemically prepared nitrate solutions with concentration similar to that found in Air GPAW (5 mM KNO_3_^−^) resulted in smaller differences with respect to the control. In particular, differences of 39.3%, 31.7%, 9.5% and 13.4% at, respectively, 0.06, 0.16, 0.28, and 0.7 µmol m^−2^ s^−1^ (significant changes at 0.06 and 0.16 µmol m^−2^ s^−1^; *p* < 0.0001) were observed. No significant improvement was detected in LFR conditions (>1 µmol m^−2^ s^−1^) (Figure 4a).

The effect of Air GPAW under varying light intensities on the ER of *N. tabacum* seeds followed the same trend (Figure 4b). Under VLFR, Air GPAW showed positive mean differences of 58%, 23.4%, and 11.3% with respect to the control under 0.06, 0.16 and 0.27 µmol m^−2^ s^−1^ light conditions, respectively; the results at 0.06 and 0.16 µmol m^−2^ s^−1^ were statistically significant (*p* < 0.0001). At higher light intensities (LFR), the effects were not statistically significant. Chemically prepared nitrate solutions only caused a small change in ER at 0.06 µmol m^−2^ s^−1^ (mean difference of 14.1%; *p* < 0.05), but no further significant changes were detected across higher light intensities.

#### 2.1.2. He/O_2_ GPAW Impact on *N. tabacum* Photo-Dependent Dormancy Alleviation

He/O_2_ GPAW also displayed notable effects on dormant *N. tabacum* seeds across the full range of light intensities. As shown in Figure 4a, there was a significant increase in the percentage of TR. Under VLFR conditions at 0.06, 0.16 and 0.28 µmol m^−2^ s^−1^, there were significant increases in TR of 36.9%, 31% and 54%, respectively. Under LFR conditions, the differences were smaller but remained significant at 1.46, 3.99 and 58.1 µmol m^−2^ s^−1^ with increases in the mean TR by 45.3%, 36.3% and 21.1%, respectively. In He/O_2_ GPAW, the main long-living reactive species found in the solution was H_2_O_2_. However, the use of chemically prepared H_2_O_2_ solutions (300 µM) led to no significant improvement with respect to the control, although the He/O_2_ GPAW led to clear improvements in TR performance (Figure 4a).

The same pattern of results was demonstrated 5 days post imbibition for ER (Figure 4b). He/O_2_ GPAW under VLFR light conditions significantly increased the ER mean (*p* < 0.05) by 23.6%, 16.6% and 17.5% at 0.06, 0.16 and 0.28 µmol m^−2^ s^−1^, respectively, in contrast to the control. Under VLFR conditions, the He/O_2_ GPAW treatment led to significant ER improvements in contrast to H_2_O_2_. Under LFR conditions >1 µmol m^−2^ s^−1^, no significant differences were detected between He/O_2_ GPAW and either the control or H_2_O.

### 2.2. Effect of GPAW Treatment on the Relative Gene Expression of N. tabacum under Varying Light Conditions

**Figure 5:**
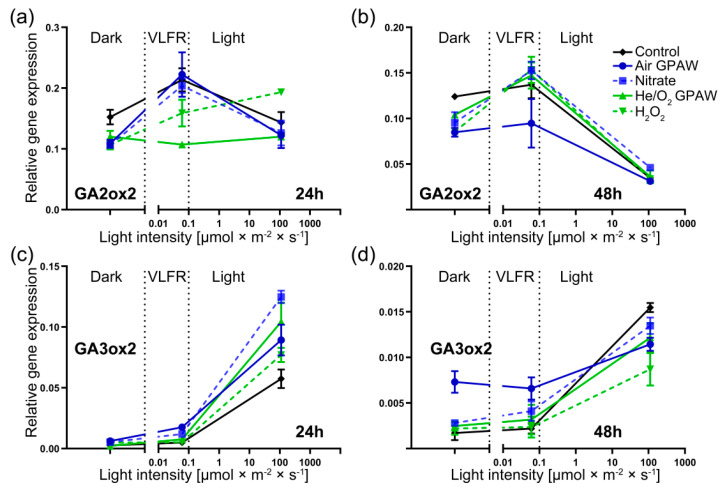
Relative gene expression of freshly harvested *Nicotiana tabacum* seeds displaying photo-dependent dormancy and germination responses in three different continuous light conditions (darkness (0 µmol m^−2^ s^−1^), VLFR-intensity white light (0.05 µmol m^−2^ s^−1^) and LFR-intensity white light (111 µmol m^−2^ s^−1^)) at 2 time points (24 and 48 h). Seeds were incubated at 20 °C with 5 treatments: Air GPAW, He/O_2_ GPAW, Nitrate (5 mM KNO_3_^−^), H_2_O_2_ (300 µM), and water. (**a**,**b**) Gene expression of *GA2ox2* at 24 and 48 h, respectively. (**c**,**d**) Gene expression of *GA3ox2* at 24 and 48 h, respectively.

The expression of genes closely associated with seed dormancy were analysed across three distinct light conditions (darkness and VLFR- and LFR-intensity white light). Figure 5a,b shows how the *GA2ox2* relative gene expression differed across treatment groups 24 and 48 h post imbibition. In total darkness, the expression of *GA2ox2* was low, with no statistically significant differences between treatments.

Under VLFR light conditions, the expression of *GA2ox2* was higher than that in darkness. No significant difference was exhibited in nitrogen-containing treatments at 24 h (Air GPAW and Nitrate); however, the He/O_2_ GPAW treatment significantly reduced *GA2ox2* expression by 49.9%, and H_2_O_2_ showed a 25.7% reduction (Figure 5a). At 48 h post imbibition under VLFR light conditions, all treatments apart from Air GPAW displayed similar expression levels to the control; Air GPAW reduced expression by 30.9% (*p* < 0.05). Under full light, *GA2ox2* expression was down-regulated (48 h) and showed no significant differences between treatments and the control except for H_2_O_2_ at 24 h (significant increase of 34.7%; *p* < 0.05) (Figure 5b).

For *GA3ox2* in darkness, there was no difference in relative gene expression between any treatment groups at 24 h; however, after 48 h of imbibition, Air GPAW increased *GA3ox2* transcript abundance by 326.1% (*p* < 0.0001) with respect to the control and by 160.7% (*p* < 0.0001) with respect to the nitrate solution. Similarly, in VLFR conditions, only Air GPAW treatment increased the expression of *GA3ox2* at 48 h by 200.7% (*p* < 0.05) with respect to the control and by 60.6% with respect to the nitrate solution (*p* < 0.05) (Figure 5c,d). There was no difference in the expression of *GA3ox2* in either dark or VLFR light conditions at both 24 and 48 h post imbibition for all other treatments.

Under full light, the various treatments resulted in significant differences at both time points. After 24 h, the nitrate solution elicited the highest expression level of *GA3ox2* with respect to the control (+117.6%; *p* < 0.05), He/O_2_ GPAW caused an increase of 81.7 % (*p* < 0.05), Air GPAW caused an increase of 55.6% (*p* < 0.05), and H_2_O_2_ caused an increase of 34.6% (*p* < 0.05) (Figure 5c). Therefore, Air GPAW showed a significant lower increase in GA3ox2 expression levels than the nitrate solution (28.5% lower; *p* < 0.05), whilst He/O_2_ GPAW demonstrated a 35.4% increase with respect to H_2_O_2_ (*p* < 0.05) (Figure 5c). After 48 h in full light, only the H_2_O_2_ treatment caused a significantly lower relative expression of *GA3ox2* in *N. tabacum* seeds in contrast to the control (H_2_O_2_: −43.7%) (Figure 5d).

**Figure 6:**
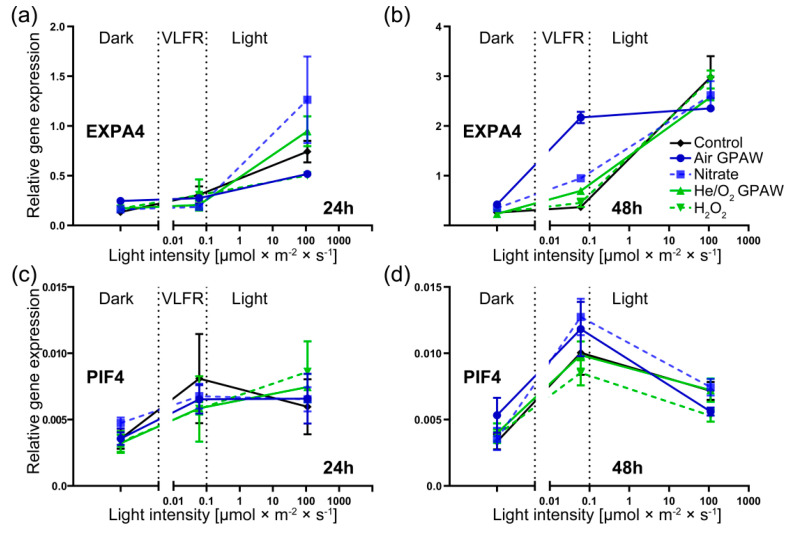
Relative gene expression of freshly harvested *Nicotiana tabacum* seeds displaying photo-dependent dormancy and germination responses in three different continuous light conditions (darkness (0 µmol m^−2^ s^−1^), VLFR-intensity white light (0.05 µmol m^−2^ s^−1^) and LFR-intensity white light (111 µmol m^−2^ s^−1^)) at 2 time points (24 and 48 h). Seeds were incubated at 20 °C with 5 treatments: Air GPAW, He/O_2_ GPAW, Nitrate (5 mM KNO_3_^−^), H_2_O_2_ (300 µM), and water. (**a**,**b**) Gene expression of *EXPA4* at 24 and 48 h, respectively. (**c**,**d**) Gene expression of *PIF4* at 24 and 48 h, respectively.

**Figure 7:**
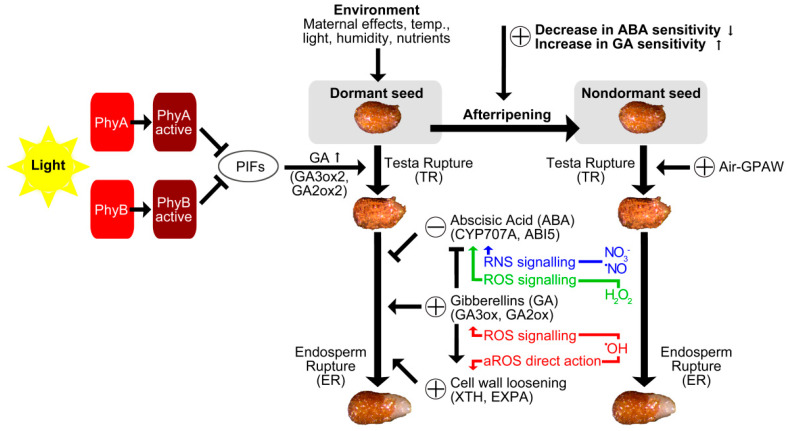
Schematic presentation of ROS and RNS signalling pathways. Phytochromes are activated and deactivated by red and far red light. Active phytochromes trigger cascades that lead to germination. Dry storage (after-ripening) reduces the dormancy status and enables seeds to complete germination under favourable conditions. Reactive oxygen species (ROS) and reactive nitrogen species (RNS) perform oxidative and nitrosative signalling to induce seed germination and release dormancy, particularly through cross-talk with plant hormones such as abscisic acid (ABA) and gibberellin (GA). Apoplastic ROS (aROS) mediates endosperm weakening and radicle growth via the direct chemical scission of cell walls [19,31].

*EXPA4* gene showed a significant increase in expression in the light conditions compared to dark or VFLR conditions across all treatment sets, particularly after 48 h of imbibition (Figure 6a,b). In total darkness, no significant difference was observed for the transcript levels for *EXPA4* among any treatment groups across both time points, and this was also the case at 24 h under VLFR light conditions. However, at 48 h post imbibition under VLFR light conditions, when no other statistically relevant differences were shown between any of the other treatment groups against the control, there was a significant increase in expression of EXPA4 in the Air GPAW treatment group (+487%; *p* < 0.0001) (Figure 6b). Furthermore, Air GPAW significantly increased relative EXPA4 expression compared to the nitrate solution (+128%; *p* < 0.0001). Under full light conditions, there were no significantly different results in EXPA4 expression at either time point. The relative expression of PHYTOCHROME-INTERACTING FACTOR 4 (PIF4) was increased in light conditions compared to darkness after 24 and 48 h, but there were no significant differences between the treatments (Figure 6c,d). We previously researched photo-dependent seeds and demonstrated that GPAW can replace *A. thaliana* seeds after-ripening to release seed dormancy [31]. The proposed interactions of light with the GPAW-derived ROS and RNS pathways are summarised in Figure 7 and referred to in the discussion.

## 3. Discussion

In positively photoblastic varieties of *N. tabacum*, such as Havana 425, germination is photo-dependent and requires light [34]. Accordingly in this study, freshly harvested mature dormant seeds did not germinate in complete darkness, but the light requirement was partly released during 12 weeks of seed after-ripening. In full light, both testa rupture (TR) and endosperm rupture (ER) reached nearly 100% and showed no differences among treatment groups (Figure 1c). This supports the understanding that the regulation of *N. tabacum* dormancy and germination is, in part, under the control of the PHYA and PHYB systems. Recent work by Dong et al. [35] demonstrated that different tobacco genotypes differ in the depth of photo-dependent dormancy and that the over-expression of NtPHYB1 prevents the induction of photo-dependent dormancy during seed maturation. *Nicotiana* species are known to have one *PHYA* gene and, due to duplications during Solanaceae genome evolution, several *PHYB* genes [35,36,37,38,39]. These light-sensing proteins regulate seed germination independently from each other, both spatially and temporally [17,40,41,42]. PHYB triggers the classical red (600–650 nm)/far red (730 nm) light-reversible control of seed germination first described by Borthwick [11] using *Lactuca sativa* Grand Rapids; this is a low fluence response (LFR) that requires a photon fluence rate of 1–1000 µmol m^−2^ s^−1^. For *A. thaliana*, it is known that PHYA breaks dormancy and induces germination via the very low fluence response (VLFR; <1 µmol m^−2^ s^−1^) in wavelengths from across the whole visible spectrum [13]. Recently, Grainge et al. [31] revealed the underlying molecular mechanisms by which GPAW releases physiological dormancy of seeds in the model plant *A. thaliana* by interacting with signalling pathways targeting gibberellin and abscisic acid metabolism, as well as the expression of downstream cell wall remodelling genes.

Our results (Figure 2 and Figure 3) showed that treatment with both Air GPAW and He/O_2_ GPAW had a clear positive influence on both TR and ER percentages under VLFR conditions. What is interesting is that we observed germination-stimulating effects from both GPAW samples used in this study, Air GPAW and He/O_2_-GPAW. Air GPAW has a significant concentration of nitrate (45 min; 4.9 mM) and a low H_2_O_2_ concentration (45 min; 47.3 µM) in contrast to He/O_2_ GPAW, which had a very low nitrate concentration (18 µM) and a relatively high H_2_O_2_ concentration (294 µM) [43]. It is therefore probable that we are looking at two distinct modes of action. We propose a working model for GPAW’s effects on tobacco seed germination in Figure 7. According to this model, the seed’s dormancy status is altered by after-ripening, which enables the completion of germination under favourable conditions while also altering sensitivity to light. A certain degree of after-ripening also increases sensitivity to GPAW treatment and thereby also enables GPAW-mediated, enhanced tobacco seed germination in darkness, which is dependent on GA biosynthesis (Figure 1 and Figure 2). This is achieved via multiple pathways triggered by the major chemical species produced in GPAW including nitrate (NO_3_^−^), nitric oxide (·NO), hydrogen peroxide (H_2_O_2_) and transient species such as hydroxyl radicals (OH·). Reactive oxygen species (ROS) signalling leads to the induction of the GA3ox genes in seeds catalysing the biosynthesis of GA. Apoplastic ROS (aROS) produced in the cell wall are involved in embryo growth and endosperm weakening. Crosstalk between reactive nitrogen species (RNS) and phytohormones plays a prominent role in seed germination.

Air GPAW showed improvement not only across a range of VLFR and LFR light conditions compared to the control but also in contrast to seeds treated with a 5 mM KNO_3_^−^ solution; the concentration of nitrate was similar to that measured in Air GPAW. The same can be said for He/O_2_-GPAW, which led to significant improvements across the whole range of light conditions in contrast to the H_2_O_2_ treatment, with a similar concentration to that measured in He/O_2_ GPAW. This is evidence that Air GPAW and He/O_2_ GPAW affect germination across light conditions in distinct ways that are also distinct from the known effects of NO_3_^−^ and H_2_O_2_, respectively. Potentially, GPAW treatments could influence germination performance by influencing mechanisms regulating dormancy that bypass the light requirement for germination, positively influencing the mechanism downstream of phytochromes (including VLFR PHYA activation) and/or affecting the physiological regulation of germination directly, e.g., by releasing the mechanical resistance imposed on the micropylar endosperm by the seed-covering layers [18,19,31,44].

Nitrate, nitric acid and nitric oxide (⋅NO) are well-documented as compounds to attenuate dormancy and increase the germination of photo-dormant seeds, a phenomenon that seemingly depends on crosstalk with the abscisic acid (ABA) signalling network. In the case of Air GPAW, bypassing the light requirement for germination cannot simply be attributed to nitrate signalling through the NLP8, CYP707A2 mechanism described for *A. thaliana* [45], because germination performance across the full range of light availability was significantly lower for the nitrate treatment in this study (Figure 4). There were also significant differences between the two treatments across the GA biosynthesis pathway (Figure 5). GA has been shown to break the photo-dependent dormancy of *N. tabacum* seeds [46]. The GA requirement for tobacco seed germination in darkness was also released during after-ripening [34]. The reversible LFR regulation of dormancy and germination operates through the conversion of phytochromes from their inactive (Pr) to their active form (Pfr) via light interaction [40]. It has been shown in *A. thaliana* that light-activated phytochromes (A and B) interact with and promote the degradation or repression of negative germination regulators such as PHYTOCHROME-INTERACTING-FACTOR 1 (PIF1), REVEILLE 1 (REV1) and REVEILLE 2 (REV2) [12]. PIF1, REV1 and REV2 inhibit seed germination by repressing the gibberellin 3-oxidase genes *GA3ox1* and *GA3ox2*, two key enzymes that regulate the rate of bioactive GA biosynthesis. Furthermore, PIF1 has been shown to up-regulate the catabolic gene *GA2ox2* and two GA repressor (DELLA) genes. PHYTOCHROME-INTERACTING-FACTOR 4 (PIF4) is known to be an integrator of light and temperature cues [47]. However, our *N. tabacum* seed gene expression pattern analysis does not provide any evidence for PIF4’s involvement in the mode of action of different GPAWs. Depending on the species, plants contain a varying set of PIFs, with eight members in *A. thaliana* and a similar number in Solanaceous species [48]. Future work may focus on identifying the PIF regulators involved in mediating light responses during tobacco seed germination (Figure 7).

For Air GPAW after 48 h of imbibition, *GA2ox2* showed significant down-regulation under very low fluence light conditions and *GA3ox2* was up-regulated in both dark and very low fluence conditions (Figure 5). This indicates that Air GPAW drives changes in the transcriptome, which influence the pathways that govern GA’s regulation of dormancy. This could be due to synergistic effects and additional reactive species formed in Air GPAW. For example, air plasmas can produce ⋅NO [49], which could in turn influence germination through the N-end rule pathway [50]. Through this pathway, ⋅NO has been shown to activate germination by promoting dormancy release and reducing sensitivity to ABA [51,52]. Furthermore, short-lived molecules can cause the S-nitrosylation of the transcription factor ABI5, leading to its degradation by CULLIN4-based and KEEP ON GOING E3 ligases, thereby promoting germination [53].

Downstream of increasing active GA biosynthesis, the up-regulation of cell wall remodelling proteins (CWRP) such as EXPA and XTH would be expected, which is in agreement with our results (Figure 6). There was a clear pattern of *GA3ox2* up-regulation in full light and increased EXPA4 activity for all treatment groups. These results were in agreement with the lower puncture force observed in seeds that underwent testa rupture (and were therefore further along in the process of germination) (Figure 1b). Under VLFR conditions, there was a significant reduction in *GA3ox2* expression for all treatments, in contrast to full light, and this was also seen for *EXPA4* (Figure 6) except for Air GPAW treatment. Air GPAW demonstrated a clear up-regulation of EXPA4 expression at 48 h in contrast to all other treatments, which appeared to be due to enhanced GA accumulation because *GA3ox2* expression was also enhanced under VLFR light conditions compared to both nitrate and H_2_O. Therefore, Air GPAW might influence germination performance outside of the nitrate–NLP8–CYP707A2 mechanism, possibly through the N-end rule pathway [31,54,55]. 

He/O_2_ GPAW was most influential in VLFR light conditions by suppressing *GA2ox2* expression at 24 h in contrast to all treatment groups, including H_2_O_2_ (Figure 5a). This could have been because short-lived ROS synthesised in He/O_2_ GPAW through both primary and secondary reactions provide a more oxidative environment that triggers the down-regulation of *GA2ox2* under VLFR conditions. This idea is supported by previous work that demonstrated that H_2_O_2_ significantly down-regulated *GA2ox2* expression in *N. tabacum* [56]. H_2_O_2_ and He/O_2_ GPAW both led to an up-regulation of *GA3ox2* expression at 24 h (Figure 5c), which is in agreement with the previously reported generation of endogenous GA_3_ by H_2_O_2_ (50 mM) treatment [56]. H_2_O_2_ is believed to be a major regulator of GA biosynthesis in ROS dormancy-alleviation mechanisms. However, our results suggest that other reactive species are significant factors as well.

He/O_2_ GPAW likely increased germination performance under VLFR conditions due to ROS signalling; however, it is also possible that GPAW bypasses the requirement for light by directly promoting ER weakening—a limiting factor to seed germination in *N. tabacum* [31,44]. ROS are known to break dormancy and stimulate germination by direct action on cell walls. For example, it is known that O_2_^.−^, H_2_O_2_, and ·OH accumulate in ER during weakening and in the growing embryo to aid cell-wall weakening during seed germination [57]. GPAW directly and indirectly triggers multiple molecular mechanisms via signalling pathways. The signals created by GPAW mimic a range of environmental signals that are perceived by seeds (Figure 7). Major chemical species produced in GPAW are also naturally produced in plants and are known for their signalling roles and direct chemical actions on cell walls. In summary, this work provides physiological evidence that GPAW can remove the photo-dependency to stimulate seed germination of dormant *N. tabacum*, a common model for photo-dependent, physiologically dormant seeds. This discovery has many potential applications. Seed dormancy and its regulation is a survival mechanism essential for adapting to and colonising new environments and for sustainably intensifying agricultural production. Our results offer several exciting avenues of research into the details of the underpinning mechanisms that should be pursued further. 

## 4. Materials and Methods

### 4.1. Seed Material

Dormant *Nicotiana tabacum* L., cultivar Havana 425, seeds were produced through a fresh propagation. Plants were grown in a rotation of 28/20 °C in a 16/8 h cycle, respectively. The harvest of mature seeds took place ca. 40 d after pollination when brown capsules were mature, and then seeds were dried at 15% relative humidity (RH) before being frozen in airtight containers at −20 °C. Seeds were defrosted at room temperature 2 h prior to experiments. For after-ripening, seeds were placed within an environment of 33% RH at 20 °C for specified periods in each assay (in the headspace of a sealed container above a saturated MgCl_2_ solution).

### 4.2. Gas-Plasma-Activated Water (GPAW) Synthesis

GPAW was produced by exposing distilled water to non-equilibrium air and He/O_2_ plasmas that were sustained using a dielectric barrier discharge (DBD) reactor [31,58]. A microporous stainless-steel membrane that served as the ground electrode was used to create microbubbles in the solution, and the high voltage electrode consisted of 37 homogeneously spaced stainless-steel rods 5 mm in diameter encased in quartz tubes, which were placed beneath the ground electrode. The carrier gas flow was regulated by mass flow controllers (Alicat Scientific Inc., Tucson, AZ, USA) and was directed past the electrodes into the solution chamber as microbubbles.

For the He/O_2_ GPAW treatment, a mixture of 98% helium (BOC N4.6) and 2% oxygen (BOC N5.0) was used at a flow rate of 1 standard litre per second (SLPM); for this treatment, the reactor operated at 8.0 kV 30.1 kHz. For the air treatment, compressed air (BOC compressed industrial air grade) was used at a flow rate of 1 standard litre per second (SLPM); for this treatment, the reactor operated at 18 kV 24.3k Hz. The voltage and frequency were measured using a Tektronix P6015A high voltage probe and a TBS 1102B Digital Oscilloscope, respectively.

### 4.3. Chemical Characterisation of GPAW

The chemical characterisation of the Air and He/O_2_ GPAWs is shown in Table 1. H_2_O_2_ was quantified using a modified method first described by Eisenberg [59]. Titanium oxysulphate (75 µL) was mixed with H_2_O_2_ standards or GPAW in a 96-well transparent plate (CoStart Washington, DC, USA). This reacted with H_2_O_2_ in solution to form pertitanic acid, which could be optically detected. In particular, the substrate has a light absorption peak at 407 nm, which was recorded using a Tecan Spark 10 M plate reader (Tecan Trading AG, Männedorf, Switzerland). A standard curve with a range of 0–10 mM was used to calculate the molar extinction coefficient. 

NO_2_^−^ and NO_3_^−^ were quantified by a Griess-reagent-based method, as demonstrated by García-Robledo et al. [60]. A standard curve between 0 and 80 µM was produced to calculate the molar extinction coefficient. Absorbance at 540 nm was measured using a Tecan 10 M plate reader.

Hydroxyl radicals were quantified using terephthalic acid (4 mM), which could be detected through fluorescence (excitation 315 nm and emission at 425 nm) when hydroxylated to 2-hydroxy terephthalic acid (HTA). The reaction was buffered to pH 6.8 using a potassium phosphate buffer (20 mM), and a standard curve was produced to calculate the molar extinction coefficient. A 45 min reaction time was chosen for all experiments.

### 4.4. Germination Assays

Germination experiments were conducted in four biological replicates. Approximately 40 seeds were placed in 6 cm petri dishes with two ⌀ 5.5 cm cellulose filter papers (MN713, Macherey–Nagel, Dueren, Germany) and 1.5 mL of the specified treatment solution. For the incubation in continuous darkness, seeds were plated under a green light condition and wrapped with two sheets of aluminium foil. Germination assays in the full light were carried out under continuous white light (Panasonic versatile environmental test chamber MLR-352, light setting 4LS, fitted with OSRAM lumilux cool white 36W/840) at a fluence rate of 111 µmol m^−2^ s^−1^, as indicated in Figure 6b.

Testa rupture was visually recorded using a microscope. A crack in the dark testa with clear exposure of the endosperm determined a rupture. Endosperm rupture was counted when the radicle protruded the covering endosperm. For statistical comparisons, replicate data points were analysed through ANOVA and Tukey’s analysis. 

### 4.5. Puncture Force Analysis

Puncture force experiments were performed as described previously [44]. Imbibed seeds were cut in half, their embryos were removed, and the endospermic tissues were placed in sample holders. A rounded 0.2 mm metal pin was driven into the sample with a speed of 0.35 mm min^−1^ while force and displacement were recorded simultaneously. The micropylar endosperm was measured at 3 h (no TR) and 36 h (TR).

### 4.6. RNA Extraction and cDNA Synthesis

For each sample, 20 mg of seeds were collected at specified times, frozen in liquid nitrogen, and stored at −80 °C. Total RNA was extracted using the RNAqueous™ Total RNA Isolation Kit (Invitrogen) according to manufacturer’s instructions. Quantification and purity were measured via a Tecan 10 M plate reader, and only samples with OD ratios of at least 2.2 (260/280 nm) and 2 (260/230 nm) were used for cDNA synthesis. cDNA was synthesised from 1 µg of total RNA using a Superscript III reverse transcriptase (Invitrogen), as described by Grainge et al. [31].

### 4.7. RT-qPCR Protocol and Analysis

RT-qPCR was performed as described by Grainge et al. [31] based on the method described by Graeber et al. [61] with a slight modification. The PCR programme was: 15 min at 95 °C, followed by 50 cycles of 15 s at 95 °C, 30 s at 58 °C and 30 s and 72 °C. Three biological replicates were used for each time point. Primers used were designed using the annotated tobacco sequence of Sierro et al. [62]. The data were normalised against three reference genes (AP2, PP2A and ACT7) selected from a short list of 5 candidates genes identified from the work of Dekkers et al. [63]. The most stable genes were selected utilising refiner software. Data were analysed using the (1 + EAveragePerAmplicon) − CT Individual Sample approach detailed by Graeber et al. [61].

## Figures and Tables

**Table 1:** Mean chemical concentrations ± SEM in gas-plasma-activated water during non-thermal atmospheric gas plasma reaction.

Gas	Chemical	Reaction Time (min)				
	(µM)	10	15	30	45	60
He/O_2_	H_2_O_2_	-	-	180.4 ± 7.2	294.9 ± 18.4	387.7 ± 24.5
	·OH	16.7 ± 3.3	25.5 ± 2.9	55 ± 4.0	80.0 ± 2.9	-
	NO_3_^−^	-	-	22.7 ± 3.6	18.1 ± 3.8	15.7 ± 1.9
	NO_2_^−^	-	-	0	0	0
Air	H_2_O_2_	-	-	47.8 ± 3.3	33.3 ± 2.0	30.2 ± 0.2
	·OH	14.5 ± 3.3	23.1 ± 2.3	42.7 ± 5.2	54.3 ± 4.7	-
	NO_3_^-^	-	-	3420.7 ± 103.5	4948.6 ± 74.5	6191.1 ± 101.2
	NO_2_^-^	-	-	56.5 ± 2.5	47.3 ± 1.7	35.4 ± 4.0

## Data Availability

All data presented or analysed in this published article are available online through figshare https://doi.org/10.17637/rh.20073782 (accessed on 13 June 2022).

## Abbreviations

ABA	Abscisic acid
ER	Endosperm rupture
GA	Gibberellin
GPAW	Gas-plasma-activated water
H_2_O_2_	Hydrogen peroxide
LFR	Low fluence response
·NO	Nitric oxide
NO_3_^−^	Nitrate
OH·	Hydroxyl radical
PHYA/B	Phytochrome A/B
TR	Testa rupture
VLFR	Very low fluence response
